# Redox Enzymes of the Thioredoxin Family as Potential and Novel Markers in Pemphigus

**DOI:** 10.1155/2021/6672693

**Published:** 2021-04-01

**Authors:** P. Sliwiak, E. Folwarczny, D. Didona, S. Fink, C. Wiegand, E. M. Hanschmann, M. Hertl, C. Hudemann

**Affiliations:** ^1^Department of Dermatology and Allergology, Philipps University Marburg, Marburg, Germany; ^2^Department of Dermatology, Jena University Medical Center, Jena, Germany; ^3^Department of Neurology, Medical Faculty, Heinrich-Heine University Düsseldorf, Düsseldorf, Germany

## Abstract

Pemphigus vulgaris (PV) is a severe autoimmune blistering disease affecting both skin and mucous membranes. Its pathogenesis is related to IgG autoantibodies primarily targeting the cellular adhesion protein desmoglein (Dsg) 3, one of the major desmosome components. Impaired redox regulation is considered a major player in the pathogenesis of autoimmune diseases such as pemphigus by enhancing inflammation and breakdown of immunological tolerance by structural protein modifications. Despite many recent advances, local and systemic redox profiles that characterize the immune response in pemphigus are virtually unknown but potentially crucial in further advancing our understanding of redox-dependent modifications that eventually lead to clinical manifestation. Here, we have analyzed the individual expression pattern of four major redox enzymes that are members of the thioredoxin (Trx) fold superfamily (peroxiredoxins (Prxs) 1 and 4, glutaredoxin (Grx) 2, and Trx1) in serum and PBMCs as well as their distribution in the skin of pemphigus patients compared to healthy controls. We show that in groups of five pemphigus patients, Prx1 is upregulated in both serum and PBMCs, while its epithelial distribution remains within the spinous epithelial layer. Expression of Grx2 and Prx4 is both reduced in serum and PBMCs, while their distinct and similar expression in the skin changes from an even distribution throughout the basal layer (healthy) to ubiquitous nuclear localization in pemphigus patients. In PV patients, Trx1 is secreted into serum, and cellular distribution appears membrane-bound and cytosolic compared to healthy controls. We furthermore showed that a 3D *ex vivo* human skin model can indeed be used to reproduce similar changes in the protein levels and distribution of redox enzymes by application of cold atmospheric plasma. Deciphering the relationship between redox enzyme expression and autoimmunity in the context of pemphigus could be critical in elucidating key pathogenic mechanisms and developing novel interventions for clinical management.

## 1. Introduction

Various autoimmune diseases are characterized by abnormalities at the tissue and cellular levels, as well as an impaired redox regulation affecting protein, DNA, and signaling. Pemphigus encompasses a group of severe autoimmune blistering diseases related to IgG autoantibodies targeting adhesion molecules in the epidermis such as desmoglein (Dsg) 1 and 3 [1]. Treatment regimens so far focused on nonspecific immune suppression and antigen-specific T and B cells are currently the focus of scientific efforts to individualize strongly needed personal treatment options [2]. Oxidative modifications caused by reactive oxygen species (ROS) such as hydroxyl radicals or the second messenger hydrogen peroxide affect the active site of proteins potentially leading to a loss of structural or functional activity [3]. Posttranslational modifications may form neoantigenic self-peptides, which are then presented to autoreactive T and B cells that have escaped negative selection in the thymus. Subsequently, autoreactive T and B cells infiltrate a given tissue potentially leading to the development of an autoimmune response [4]. Recent data showed that also reduced ROS production by T cells in rheumatoid arthritis leads to dysregulated lipogenesis and enhanced tissue infiltration putting superoxide radicals into a hallmark position connecting protective versus autoaggressive T cell immunity [5]. In autoimmune blistering diseases such as pemphigus vulgaris (PV), relatively little is known about a correlation between oxidative distress and the clinical outcome. A general lower total antioxidant capacity and a positive correlation to the pemphigus-associated alleles HLA DRB1∗0402 and DQB1∗0503 were found in PV patients compared to control [6, 7]. Increased ROS production correlates with higher enzymatic activity (glutathione peroxidase, catalase), especially by activated neutrophils, and in turn reduces concentrations of antioxidant vitamins A and E [8, 9]. Lipid peroxidation can be found as an indirect result of increased amounts of conjugated dienes and has been implicated in several autoimmune disorders, including systemic lupus erythematosus and PV [10, 11]. Cells have developed a set of protein thiol-disulfide oxidoreductases, such as the thioredoxin family, to regulate oxidative and protein function. Members like glutaredoxins (Grx), thioredoxins (Trx), and peroxiredoxins (Prx) are essential for the redox regulation of cellular functions by sensing hydrogen peroxide and catalyzing the reduction and partly also oxidation of protein thiol groups. Interestingly, they display a distinct cellular distribution pattern, including the active release to the extracellular space. They either function intracellularly as regulators of the “redoxome” or are secreted to function systemically, e.g., as chemokines (Trx [12]), paracrine factors (Trx [13]), or natural killer cell activator (Prx [14]). Trx1, Grx2, and Prx1 and Prx4 were shown to play a role in cell migration, inflammation, and wound healing, for instance, via regulation of the transcription factor NFkB or TLR4 signaling [15]. However, the impact of these mentioned thiol-based enzymatic systems in PV and their therapeutical potentials are largely unknown.

In this study, we performed an exploratory analysis to correlate the aforementioned redoxins systemically in PBMCs as a pool of migrating inflammatory cells and serum representing the main compartment containing antigen-specific autoantibodies. We then analyzed protein distribution in the site of inflammation in PV patients compared to healthy controls. We hypothesized that, induced by the disease state, we can observe a protein-specific distribution pattern which will allow further mechanistic analysis contributing to novel treatment development.

Additionally, we asked whether the found *in vivo* results can be mirrored in an *ex vivo* system to develop novel tools to further characterize redoxins as possible disease-specific markers and to analyze their specific contribution to a disturbed oxidative microenvironment in the skin. We therefore investigated altered enzyme distribution by immunohistochemistry in a 3D skin model treated with cold atmospheric plasma (CAP) compared to control. Plasma medicine as a field of applied redox biology is gaining increasing interest providing a multitude of dermatological applications [16]. Novel studies present promotion of wound healing by CAP via the induction of reepithelialization, neovascularization, and inflammatory response [17, 18], which in turn might introduce redox systems as novel treatment targets or markers during previously unrecognized local PV treatment regimes. We therefore asked whether CAP with unknown cause-and-effect mechanisms is able to restore or induce skin-located enzymatic systems such as the Trx, Grx, or Prx system *ex vivo*.

## 2. Materials and Methods

### 2.1. Patients and Sample Isolation

Each study participant gave written consent prior to inclusion in the study, which was approved by the Ethics Committee of the Medical Faculty of Philipps University, Marburg (AZ 63/19). The study was conducted according to the Declaration of Helsinki Principles. Peripheral blood mononuclear cells from human subjects were isolated from citrate-phosphate-dextrose-adenine- (CPDA-) treated blood samples and stored in liquid nitrogen as previously described [19]. Punch biopsies from lesional skin were obtained and fixed in paraformaldehyde, embedded in paraffin, and further processed for immunohistochemical analysis of the inflammatory infiltrate. Healthy skin samples were quickly embedded in O.C.T. Compound (Tissue-Tek) and stored at -80°C until further analysis.

### 2.2. Detection of Anti-Dsg IgG

The presence of IgG autoantibodies against Dsg1 or Dsg3 in blood of PV patients and HC was evaluated by anti-Dsg1- and anti-Dsg3-ELISA (Euroimmun, Lübeck, Germany) according to the manufacturer's protocol.

### 2.3. Preparation of 3D Skin Models and CAP Treatment

3D skin models were cultured according to Wiegand et al. [20]. In brief, normal human epidermal fibroblasts (NHDF, Promocell, Germany) were cultured in Dulbecco's modified Eagle's medium (DMEM, Promocell) supplemented with 5% fetal bovine serum (Promocell), 1% gentamycin (Life Technologies, USA), and human epidermal growth factor (5 ng/mL) at 37°C in a 5% CO_2_ atmosphere. NHDF were seeded into 12-well inserts (Greiner Bio-One, Germany) with rat tail collagen solution (Fraunhofer IGB, Germany) mixed at a ratio of 1 : 1 with gel neutralizing solution (Fraunhofer IGB, Germany) at a final concentration of 1 × 10^5^ cells/mL and incubated for 15 min at 37°C and in a 5% CO_2_ atmosphere to ensure the regeneration of the matrix before DMEM with 10% FCS, and 1% gentamycin was added. Normal epidermal keratinocytes (NHEK, Promocell, Germany) were cultured in keratinocyte growth medium with low BPE (Pelobiotech, Germany) at 37°C in a 5% CO_2_ atmosphere. NHEK were added on top of the dermis equivalent after 24 hours at a concentration of 1 × 10^6^ cells/mL and incubated in submerse medium (keratinocyte basal medium 2 with 5 *μ*g/mL insulin, 10 *μ*g/mL transferrin, 0.004 mL/mL BPE, 0.125 ng/mL epidermal growth factor, 0.33 *μ*g/mL hydrocortisone, 0.39 *μ*g/mL epinephrine, 0.06 mmol/L CaCl_2_, 5% FCS, and 1% gentamycin). After 7 days, 3D skin models were raised to the medium-air-interface and medium was changed to DMEM+HAMsF12 (ratio 1 : 1), 5% FCS, 1% gentamycin, 10 ng/mL hEGF, 0.33 *μ*g/mL hydrocortisone, 10^−4^ M adenine, 5 *μ*g/mL insulin, 5 *μ*g/mL transferrin, 2 × 10^−7^ M triiodothyronine, and 1.88 mM CaCl_2_. After 12 days, the models were fully differentiated. CAP treatment was carried out with an experimental microplasma device using air as process gas [21]. 3D skin models were treated for 40 s at a distance of 1 mm. Thereafter, skin models were incubated for 24 h at 37°C and in a 5% CO_2_ atmosphere before being transferred to 4% formalin solution for histology. Untreated 3D skin models were used as controls.

### 2.4. Immunohistochemical Analysis

Formalin-fixed tissue was paraffin- or O.C.T. compound-embedded, and 3 *μ*m thick sections were stained with *hematoxylin and eosin* (*HE*). HE-stained sections were microscopically assessed, and random images were collected under 10x objective (Supp. Figure 2). For histological analysis, sections were rehydrated with phosphate-buffered saline (PBS) twice for 5 min, treated with 3% H_2_O_2_ for 10 min to quench endogenous peroxidase activity, and washed with PBS twice again. Nonspecific binding was blocked with 10% fetal calf serum (FCS) for 1 h. Slides were incubated overnight at 4°C or for 1 h at room temperature with anti-mouse/human Prx1 and 4 (Abcam, Cambridge, UK), Trx1 (Abcam), and Grx2 [22], diluted 1 : 200. Negative controls were carried out by omitting the primary antibody. After three washes (5 min each) in PBS, the slides were incubated with biotinylated anti-rabbit IgG (Dako, Glostrup, Denmark) for 1 h at room temperature, diluted 1 : 500. The sections were washed three times, conjugated with streptavidin peroxidase (Abcam) for 30 min at room temperature. Following three additional washing steps, sections were incubated with the substrate aminoethyl carbazole (AEC, Invitrogen, Karlsruhe, Germany) for 5 min at room temperature, counterstained with Mayer's hematoxylin and mounted with solvent-free medium Fluoromount with DAPI (Thermo Fischer Scientific, Darmstadt, Germany).

### 2.5. Protein Quantification and Western Blotting

Total protein content from PBMCs was quantified by a Lowry assay (DC protein assay, Bio-Rad Laboratories, Hercules, CA). After a 5 min incubation with 100 mM DTT at room temperature followed by brief boiling, cell lysates from PBMCs (10 *μ*g/sample) or serum (2 *μ*L/well) were cleared by centrifugation. Next, samples were applied to 12% acrylamide sodium dodecyl sulfate (SDS) gels and transferred to nitrocellulose membranes (Millipore, Billerica, USA) by electroblotting (Thermo Fisher Scientific). After blocking in 5% bovine serum albumin (BSA) in PBS containing 0.05% Tween 20, membranes were probed with one of the following antibodies overnight at 4 degrees: polyclonal rabbit anti-human Prx1, Prx4, Trx1 (Abcam), or Grx2 [22]. Next, detection was done with goat anti-rabbit-IgG-horse-radish peroxidase conjugate (Dako/Agilent, Santa Clara, USA) and developed using a chemiluminescent HRP substrate (Millipore) by a western blotting imaging system (Peqlab, Erlangen, Germany).

### 2.6. Statistical Analysis

Statistical analysis was performed using GraphPad Prism 6.02 (GraphPad Software Inc., La Jolla, USA). Cumulative data are displayed as box plots with median. For group comparisons, a two-tailed nonparametric Mann-Whitney *U* test was applied. Differences between the groups were considered statistically significant at *p* values of <0.05.

## 3. Results

For quantification of the respective proteins, we performed western blot analysis on 5 random PV patients (Supp. Table 1) or healthy controls, respectively, followed by relative quantification of specific protein bands using ImageJ. Total band size as a region of interest is displayed as (total particle) area (Supp. Figure 1).

### 3.1. Trx1

**Figure 1:**
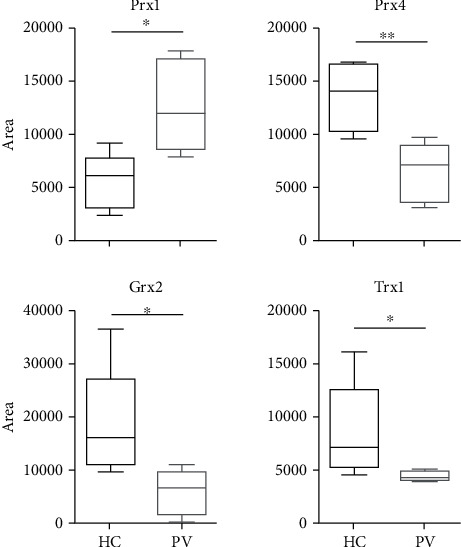
Quantification of serum protein content by western blotting on samples from healthy control (HC) or pemphigus patients (PV). Significant comparisons between HC and PV groups are indicated. ^∗^*p* < 0.05, ^∗∗^*p* < 0.01, and ^∗∗∗^*p* < 0.001. *n* = 5.

**Figure 2:**
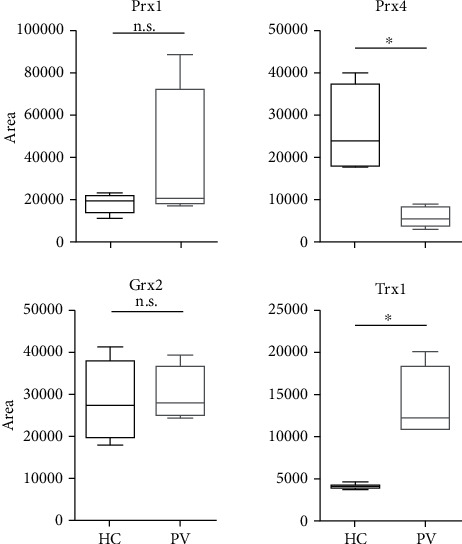
Quantification of PBMC protein content by western blotting on samples from healthy control (HC) or pemphigus patients (PV). Significant comparisons between HC and PV groups are indicated. ^∗^*p* < 0.05, ^∗∗^*p* < 0.01, and ^∗∗∗^*p* < 0.001. *n* = 5.

**Figure 3:**
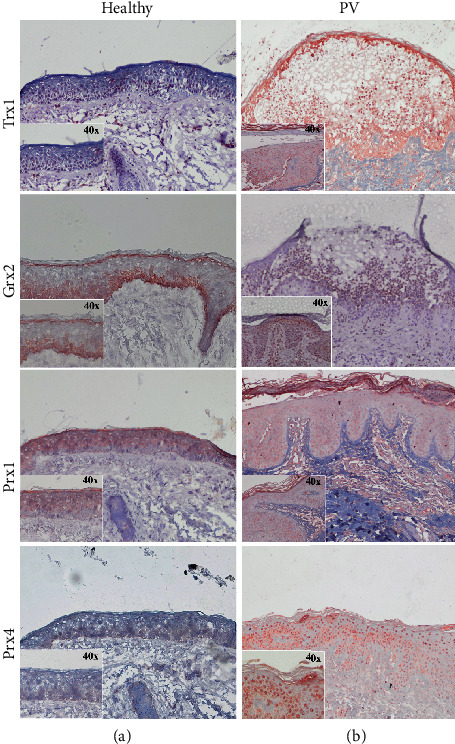
Representative immunohistochemistry on skin biopsies from 3 healthy donors (a) and from 3 PV patients. Hematoxylin staining displays in bright blue, while protein-specific staining for Trx1, Grx2, and Prx1 and 4 (indicated for each row to the left) is displayed by brown staining developed using AEC. *n* = 3.

**Figure 4:**
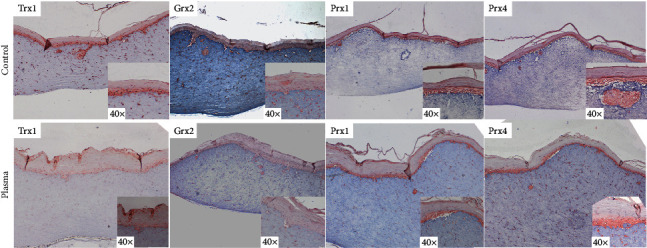
Representative immunohistochemistry on an experimental *ex vivo* skin model untreated (control) or plasma treated for 40 seconds. Hematoxylin displays in bright blue, while protein-specific staining (indicated for each row to the left) is displayed by brown staining developed by AEC.

Significantly higher protein levels of Trx1 were found in PV serum (median 12220 ± 35%SD) compared to HC (median 4296 ± 7%SD) (^∗^*p* = 0.0286). In PBMCs, however, protein concentration was found to be decreased (median 4435 ± 11%SD) compared to HC (median 7280 ± 63%SD) (*p* = 0.08) (Figures 1 and 2). A clear staining of Trx1 was observed in the skin of PV patients, in particular in the polymorphonuclear cell infiltrate present in the suprabasal blister. A distinct intercellular staining close to the blister formation was also found; Trx1 expression was rather weak in the skin of healthy donors (Figure 3). In the *ex vivo* skin model, strong Trx1 levels were found particularly in the fibroblasts of the dermal layer as well as in basal keratinocytes in the epidermal stratum. CAP treatment (plasma) however distinctly reduced Trx1 compared to the untreated control (Figure 4).

### 3.2. Grx2

Grx2 is mainly located in the mitochondria; however, various isoforms restricted to specific tissues (testis) or the cytosol present this enzyme as a multifunctional intracellular player during oxidative dysbalance such as in PV [23]. In PBMCs, a significant reduction of Grx2 from PV patients compared to HC was found (median 7136 ± 59%SD to 16651 ± 64%SD) (^∗^*p* = 0.019). However, serum levels of Grx2 remained unaltered between PV and HC (median 28083 ± 64%SD to 27491 ± 35%SD). In contrast to Prx1 but similar to Prx4, Grx2 in healthy skin biopsies was found to be distinctively expressed in the basal layer with decreasing concentration in the stratum spinosum, while in PV patients, it was moderately expressed only in the polymorphonuclear cell infiltrate and the nuclei of epithelial cells. *Ex vivo*, a ubiquitous nuclear expression in the epidermal stratum as well as in single keratinocytes was detected which was not affected by CAP treatment.

### 3.3. Prx1

The mainly cytosolic Prx1 was found to be increased significantly in PBMCs from PV (median 11897 ± 37%SD) compared to HC (median 6166 ± 41%SD) (^∗^*p* = 0.0159). Serum levels of Prx1 however did not differ much between PV (median 21099 ± 161%SD) and HC (median 19215 ± 24%SD). Prx1 is faintly homogeneously expressed in the epidermis of healthy controls excluding the stratum basale, while in PV patients, the protein appears to be membrane-bound. In the 3D skin model, neither Prx1 nor Prx4 are expressed if left untreated; however, increased amounts of Prx1 and Prx 4 can be detected, both in fibroblasts and keratinocytes of the basal layer, after 40 s of CAP treatment.

### 3.4. Prx4

The ubiquitous Prx4 is mainly localized in endoplasmic reticulum and becomes secreted during inflammatory conditions [24, 25]. Here, in PBMCs derived from PV patients (median 7063 ± 38%SD), a significant decrease compared to HC (median 13926 ± 23%SD) was found (^∗^*p* = 0.0159). Along this line, levels of serum Prx4 that are significantly decreased in PV (median 5555 ± 42%SD) compared to HC were found (median 24045 ± 43%SD) (^∗^*p* = 0.0286). Prx4 is faintly found in the basal epidermal keratinocytes from healthy skin, comparable to Grx2, whereas it highlights markedly the nuclei of the keratinocytes in PV skin.

## 4. Discussion

In the present study, we for the first time systemically analyzed redox enzyme distribution and secretion in pemphigus patients. In order to differentiate between direct inflammatory contribution and disease-specific antibody compartments, we analyzed PBMCs and corresponding serum protein distribution. We found distinct disease-dependent expression changes depending on the compartment analyzed compared to the healthy control. The physiological consequence so far is unknown; however, our study suggests that Trx1 can be regarded as a potential marker for autoimmune bullous skin diseases, while systemic loss of Prx4 and Grx2 indicates a beneficial therapeutic impact by external CAP application, resulting in a reactivation of oxidative eustress in pemphigus.

CAP effects on human cells and tissues can be observed on different levels. The primary target structure is the cell membrane with its lipids and embedded receptor proteins and enzymes. Lipid peroxidation and modification of the cell adhesion molecules were observed after CAP treatment leading to altered cell migration and signal transduction. As CAP sources are operated at ambient pressure in contact with air, large amounts of reactive oxygen and nitrogen radicals (RONS) are generated, such as atomic oxygen, ozone, superoxide, hydroxyl radicals, nitric oxide, and hydrogen peroxide. The reactive molecules reach the cell through diffusion processes, but they can also be induced directly in the cell. UV radiation and free radicals can continue to affect the DNA and thus precede changes in cell proliferation or induction of apoptosis. Overall, outcomes depend on the plasma dose and treatment time. Consequently, both stimulating and damaging effects are possible [26]. Results show that CAP treatment is well tolerated as long as it is kept short [20, 27]. So far, research concentrated on stimulatory effects on eukaryotic cells promoting faster cell proliferation and enhanced angiogenesis [28] or activation of cytokines and growth factors has been reported with the potential to shorten the wound healing process [29]. Here, we have used CAP in an experimental *ex vivo* setting to potentially counteract redox dysbalance in pemphigus PBMCs by inducing or reactivating oxidative eustress.

Trx1 was originally characterized as a substrate for ribonucleotide reductase [30] and was later identified extracellularly, highlighting the importance of systemically available redox enzymes independent of an intracellular reducing system [31]. Secretion of Trx1 follows “unconventional protein secretion pathways” [32], and elevated Trx plasma levels were already identified in several diseases such as rheumatoid arthritis [33] or pancreatitis [34]. Systemically applied Trx1 was found to be effective, for example, in mouse models of autoimmune diabetes, by reduction of diabetes onset and experimental autoimmune myocarditis by suppression of leukocyte chemotaxis [35, 36]. It is proposed to beneficially affect disease onset by either scavenging ROS, reduction and regulation of extracellular thiol switches, or modulation of receptor-mediated signaling cascades [37]. In this study, we show that serum Trx1 is indeed increased in PV patients compared to control, while PBMCs display significantly reduced protein concentrations. Extracellular Trx1 can potentially be utilized as a serum marker. This is strengthened by an increased local formation of Trx1, especially in infiltrating lymphocytes. To further specifically apply this statement onto pemphigus vulgaris, additional analysis of related autoimmune bullous skin disorders (AIBDs) such as bullous pemphigoid or mucous membrane pemphigoid is necessary [38]. Here, CAP treatment leads to a local decrease of Trx1. Since Trx1 in general is considered beneficial for wound healing [39], it would here be intriguing to see if CAP-induced local loss of Trx1 indeed leads to a Trx1 secretion in this model and *in vivo*, therefore increasing systemically available Trx1 to counteract local oxidative dysregulation found in epidermal erosions in pemphigus.

Grx2 in general appears to be decreased in PV samples, pointing at a potential therapeutical application which has been shown in other inflammatory diseases [40]. In healthy tissue, Grx2 was strongly associated with the stratum basale, similar to Dsg3 and another desmosomal protein, desmocollin 3. Here, it is tempting to further analyze the direct correlation between Grx2 and Dsg3 or desmocollins, respectively, especially since these desmosomal adhesion proteins were found to work in a redox-dependent manner [41]. Grx2 can be found in plasma [42]; whether it can however be actively secreted by cells is to date under discussion. CAP treatment did not visually affect Grx2 concentration; however, enzymatic activity would be tempting to analyze in upcoming studies.

Biological functions of extracellular Prxs are subject of ongoing studies, especially in regulation of inflammation; however, Prxs have been described as DAMP/PAMP molecules and therefore in TLR4 signaling and as NK cell activating factors [43]. Here, we found antagonistic expression patterns regarding Prx1 and 4. While in serum and PBMCs the Prx1 level is higher in PV samples, Prx4 protein levels are reduced. Locally however, PV sections were strongly positive for Prx4 with regard to epithelial cells compared to healthy controls. Current literature states indeed that tissue or blood cells with high basal or upregulated cellular expression of Prx1 or 4 are most likely a source for circulating protein in response to a changed redox environment such as in autoimmune diseases [44, 45]. To what extend each enzyme functions as a pro- rather than an anti-inflammatory agent during blister formation in PV remains to be elucidated. In the 3D skin model, both Prx1 and 4 levels could be induced by CAP treatment, indicating that stressing cells locally does indeed result in Prx upregulation to counteract induced cellular H_2_O_2_ formation as shown for type 1 diabetes [46]. We could confirm protein induction and secretion of Prx1 and Prx4 by CAP [47], while only Prx1 was induced in pemphigus PBMCs and released into serum which is particularly intriguing in terms of CAP-induced specific redox signaling events which remain to be elucidated. Note that Prx1 was shown to be released via alternative secretion, whereas Prx4 contains a signal peptide and is released via classical protein secretion which potentially explains altered protein levels only for Prx1 in PV [48].

In conclusion, we here show the specific distribution of four major redox enzymes of the thioredoxin superfamily in AIBDs exemplified by PV serum, PBMCs, local skin analysis, and a 3D skin model treated with cold atmospheric plasma. Individual protein alterations compared to healthy control HC indicate potential disease contribution.

## Data Availability

The primary data used to support the findings of this study are included within the article. The data used to support the findings of this study are included within the supplementary information file.

## Supplementary Materials

Supplementary Materials Supp. Figure 1: exemplary flow of protein quantification analysis (Prx1) in PBMC extract from healthy control (HC) or pemphigus patients (PV). (A) Western blot analysis quantified by respective first antibodies. (B, C) Calculation and display of protein presence. ^∗^*p* < 0.05, ^∗∗^*p* < 0.01, and ^∗∗∗^*p* < 0.001. Supp. Figure 2: representative photomicrographs of skin sections from PV patients stained with hematoxylin and eosin (HE) method. Lymphohistiocytic cell infiltrate (dotted arrow) in the upper dermis and suprabasal acantholytic cleft formation (straight arrow) are highlighted. Patient 1 shows clearly a row of tombstones (upper part of the skin biopsy), a characteristic clue for pemphigus. The second patient shows a more evident infiltration below the follicular structure (dotted arrow). In the skin sample of patient 3, the acantholysis, a phenomenon due to the loss of intercellular cohesion between keratinocytes, can be clearly seen (dotted arrow). Furthermore, the massive presence of neutrophils and eosinophils in the suprabasal blister can be observed. Supp. Table 1: demographics of PV patients studied by histology, serum, and peripheral blood mononuclear cell (PBMC) analysis.
