# Supplementary material for: Redox Enzymes of the Thioredoxin Family as Potential and Novel Markers in Pemphigus
Source: Oxid Med Cell Longev. 2021 Apr 1;2021:6672693. doi: 10.1155/2021/6672693 (PMC8032527; doi:10.1155/2021/6672693)
Supplement: Supplementary Materials — Supp. Figure 1: exemplary flow of protein quantification analysis (Prx1) in PBMC extract from healthy control (HC) or pemphigus patients (PV). (A) Western blot analysis quantified by respective first antibodies. (B, C) Calculation and display of protein presence. ∗p < 0.05, ∗∗p < 0.01, and ∗∗∗p < 0.001. Supp. Figure 2: representative photomicrographs of skin sections from PV patients stained with hematoxylin and eosin (HE) method. Lymphohistiocytic cell infiltrate (dotted arrow) in the upper dermis and suprabasal acantholytic cleft formation (straight arrow) are highlighted. Patient 1 shows clearly a row of tombstones (upper part of the skin biopsy), a characteristic clue for pemphigus. The second patient shows a more evident infiltration below the follicular structure (dotted arrow). In the skin sample of patient 3, the acantholysis, a phenomenon due to the loss of intercellular cohesion between keratinocytes, can be clearly seen (dotted arrow). Furthermore, the massive presence of neutrophils and eosinophils in the suprabasal blister can be observed. Supp. Table 1: demographics of PV patients studied by histology, serum, and peripheral blood mononuclear cell (PBMC) analysis. [file 6672693.f1.zip › Sliwiak et al. Supplementary_rev.docx]

**Redox enzymes of the thioredoxin family as potential and novel markers in pemphigus**

- **Supplementary -**

Sliwiak P. ^1^, Folwarczny E. ^1^, Didona D. ^1^, Fink S. ^3^, Wiegand C. ^3^, Hanschmann E.M. ^2^, Hertl M. ^1^, Hudemann C. ^1, *^

^1^ Department of Dermatology and Allergology, Philipps University Marburg, Marburg, Germany

^2^ Department of Neurology, Medical Faculty, Heinrich-Heine University Düsseldorf, Düsseldorf, Germany

^3^ Department of Dermatology, Jena University Medical Center, Jena, Germany

*** Correspondence**

Christoph Hudemann, PhD

e-mail: [christoph.hudemann@staff.uni-marburg.de](mailto:christoph.hudemann@staff.uni-marburg.de)

phone: (+49) 6421 58 64823

Total word count: 274

Figures: 2

Tables: 1

*Keywords:* redox, regulation, RONS, thioredoxin, pemphigus vulgaris, autoimmunity

**
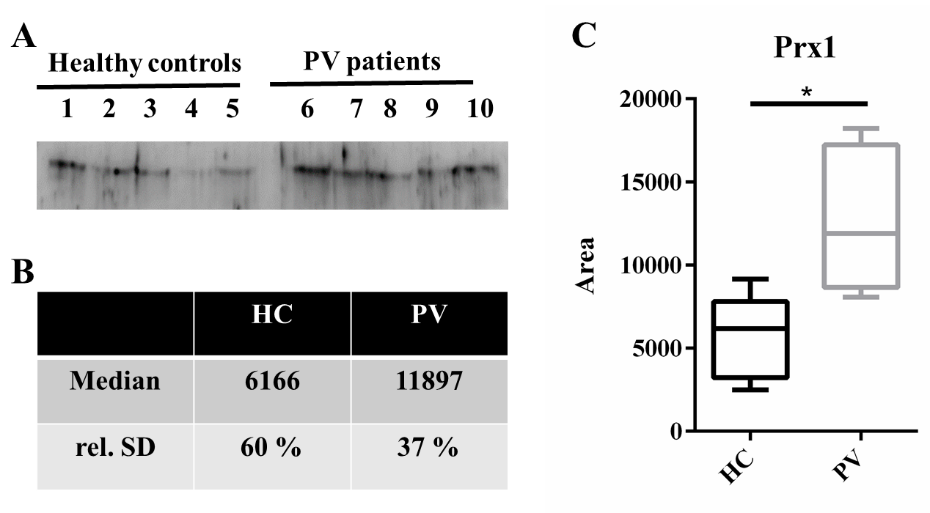
**

**Supp. Figure 1 – Exemplary flow of protein quantification analysis (Prx1) in PBMC extract from healthy control (HC) or pemphigus patients (PV). (A)** Westernblot analysis quantified by respective first antibodies. **(B,C)** Calculation and display of protein presence. **p* < 0.05, ***p* < 0.01, ****p* < 0.001.


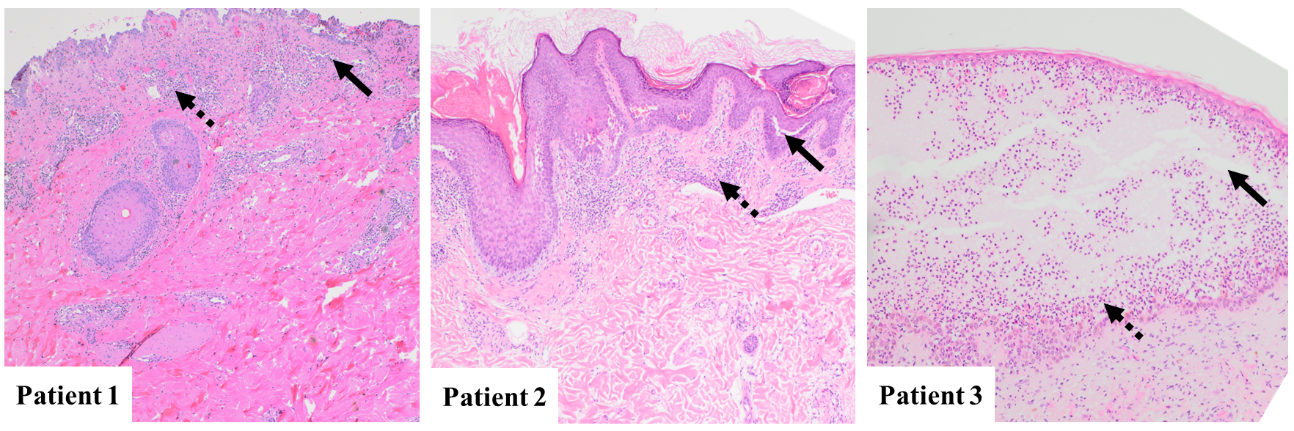


**Supp. Figure 2 – Representative photomicrographs of skin sections from PV patients stained with hematoxylin and eosin (HE) method.** Lympho-histiocytic cell infiltrate (dotted arrow) in the upper dermis and suprabasal acantholytic cleft formation (straight arrow) are highlighted. Patient 1 shows clearly a row of tombstones (upper part of the skin biopsy), a characteristic clue for pemphigus. The second patient shows a more evident infiltration below the follicular structure (dotted arrow). In the skin sample of patient 3 the acantholysis, a phenomenon due to the loss of intercellular cohesion between keratinocytes, can be clearly seen (dotted arrow). Furthermore, the massive presence of neutrophils and eosinophils in the suprabasal blister can be observed.


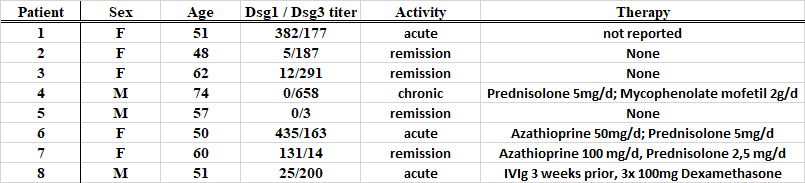


**Supp. Table 1 – Demographics of PV patients studied by histology, serum and peripheral blood mononuclear cells (PBMC) analysis.**
