# Supplementary material for: Redox Enzymes of the Thioredoxin Family as Potential and Novel Markers in Pemphigus
Source: Oxid Med Cell Longev. 2021 Apr 1;2021:6672693. doi: 10.1155/2021/6672693 (PMC8032527; doi:10.1155/2021/6672693)
Supplement: Supplementary Materials — Supp. Figure 1: exemplary flow of protein quantification analysis (Prx1) in PBMC extract from healthy control (HC) or pemphigus patients (PV). (A) Western blot analysis quantified by respective first antibodies. (B, C) Calculation and display of protein presence. ∗p < 0.05, ∗∗p < 0.01, and ∗∗∗p < 0.001. Supp. Figure 2: representative photomicrographs of skin sections from PV patients stained with hematoxylin and eosin (HE) method. Lymphohistiocytic cell infiltrate (dotted arrow) in the upper dermis and suprabasal acantholytic cleft formation (straight arrow) are highlighted. Patient 1 shows clearly a row of tombstones (upper part of the skin biopsy), a characteristic clue for pemphigus. The second patient shows a more evident infiltration below the follicular structure (dotted arrow). In the skin sample of patient 3, the acantholysis, a phenomenon due to the loss of intercellular cohesion between keratinocytes, can be clearly seen (dotted arrow). Furthermore, the massive presence of neutrophils and eosinophils in the suprabasal blister can be observed. Supp. Table 1: demographics of PV patients studied by histology, serum, and peripheral blood mononuclear cell (PBMC) analysis. [file 6672693.f1.zip › Supp Table 1.pdf]

Patient	Sex	Age	Dsg1 / Dsg3 titer	Activity	Therapy
1	F	51	382/177	acute	not reported
2	F	48	5/187	remission	none
3	F	62	12/291	remission	none
4	M	74	0/658	chronic	Prednisolone 5mg/d; Mycophenolate mofetil 2g/d
5	M	57	0/3	remission	none
6	F	50	435/163	acute	Azathioprine 50mg/d; Prednisolone 5mg/d
7	F	60	131/14	remission	Azathioprine 100mg/d, Prednisolone 2,5mg/d
8	M	51	25/200	acute	IVIg 3 weeks prior, 3x 100mg Dexamethasone
